# An implicit measure of growth mindset uniquely predicts post-failure learning behavior

**DOI:** 10.1038/s41598-024-52916-5

**Published:** 2024-02-14

**Authors:** Kata Sik, Jamie Cummins, Veronika Job

**Affiliations:** 1 Department of Occupational, Economic, and Social Psychology, University of Vienna, 1010 Wächtergasse 1, Vienna, Austria; 2 Department of Experimental Clinical and Health Psychology, Ghent University, Ghent, Belgium; 3 Institute of Psychology, University of Bern, Bern, Switzerland

**Keywords:** Psychology, Human behaviour

## Abstract

Research on implicit theories of intelligence (a.k.a. intelligence mindset) has shown that endorsing a stronger growth mindset (the belief that intelligence can be improved) is adaptive in the face of difficulties. Although the theory presumes implicit processes (i.e., unaware beliefs, guiding behaviors and actions automatically), the concept is typically assessed with self-reports. In this project we brought together research on intelligence mindset with research on implicit social cognition. Harnessing recent innovations from research on implicit measures, we assessed intelligence mindsets on an implicit level with a mousetracking Propositional Evaluation Paradigm. This measure captures the spontaneous truth evaluation of growth- and fixed-mindset statements to tap into implicit beliefs. In two preregistered laboratory studies (*N* = 184; *N* = 193), we found that implicitly measured growth mindsets predicted learning engagement after an experience of failure above and beyond the explicitly measured growth mindset. Our results suggest that implicit and explicit aspects of intelligence mindsets must be differentiated. People might be in a different mindset when making learning-related decisions under optimal conditions (i.e., with ample time and capacity) or under suboptimal conditions (i.e., when time pressure is high). This advancement in the understanding of implicit theories of intelligence is accompanied with substantial implications for theory and practice.

## Introduction

Most students experience challenges and setbacks every day. Yet, students with equal abilities respond to setbacks in various ways: some respond adaptively by approaching challenges as learning opportunities, while others get discouraged and react maladaptively (e.g., avoid further challenging situations^1^). An important factor influencing students’ reactions to difficulties and failure is their implicit theory of intelligence (a.k.a. intelligence mindset): whether they believe that intelligence is malleable (i.e., a growth mindset) or rather fixed (i.e., a fixed mindset). In achievement situations, a growth mindset (vs. a fixed mindset) is associated with having more learning goals (i.e., wanting to learn from the situation) and fewer performance goals (i.e., demonstrating intelligence for its own sake^2^). People endorsing more of a growth mindset also perceive situations as more controllable [e.g.,^3^]. Therefore, in the face of obstacles or setbacks, a stronger growth mindset predicts more adaptive reactions, like challenge-seeking [e.g.,^4^], remedial action [e.g.,^5^], or effort exertion [e.g.,^3^]. These adaptive reactions, in turn, positively affect more general learning outcomes, such as academic grades [see^6^].

Implicit theories of intelligence were named “implicit” because it is assumed that “people are often unaware of these beliefs” [p. 483^7^]. The term “implicit” has been frequently used within and across various disciplines (e.g., in linguistics^8^; in philosophy^9^; or in law^10^), but its depth and nuances has remained underexplored in the field of implicit theories. As of yet, implicit theories of intelligence have been assessed almost exclusively using self-report measures^11^, where individuals select their responses to statements in a questionnaire. These measures tend to focus on capturing thoughts and judgments which are well within the awareness of participants and can be completed at the leisure of the participant [e.g.,^12^]. In other words: there is a discrepancy between intelligence mindset’s theoretical conceptualization as often unaware and fast-acting and the measurement tools used to capture this construct (which by definition require awareness and deliberation). This calls for a fresh examination using more nuanced methodological tools, particularly in the wake of the lack of replicability of many core growth mindset research findings [e.g.,^13–15^].

Social-cognitive psychology provides a range of assessment tools that can tap into the more unaware and/or fast-acting components of beliefs and attitudes, referred to broadly as “implicit measures”^16^. These measures are traditionally designed to assess automatic or unconscious beliefs and attitudes, which are often considered less directly accessible via self-reports. For instance, a meta-analysis by Greenwald et al.^17^ demonstrated that for socially sensitive topics, such as Black-White interracial behavior, the traditional implicit measures (e.g., the most widely used implicit association test, IAT) showed greater predictive validity than self-reports. This result indicates the utility of implicit measures in scenarios where self-reports may be skewed by impression management^17^.

In the present research, we propose leveraging implicit measures from social psychology to provide novel insights into implicit theories of intelligence. In traditional implicit measures, individuals typically need to react quickly to stimuli appearing on the screen, like matching words or images, without deliberating about their beliefs or attitudes. For instance, in a Black-White IAT, participants rapidly categorize faces (Black or White) and words (positive or negative) by pressing different keys. The assumption behind these tasks is that participants' underlying beliefs or attitudes influence their response speed or accuracy. Such measures have been argued to provide unique insights into the beliefs of participants beyond self-report measures [e.g.,^17–19^].

It is important to note that the “implicit” component of these measures has been the subject of extensive theoretical debate, scrutiny, and criticism (see^20^ for a canonical criticism of the term “implicit”; or^21–23^ for general overviews about the critiques). For conceptual clarity, we use the term “implicit” in the current paper to refer to responses which are emitted quickly and which are influenced by the beliefs of the individual, occurring without the individual’s awareness. Notably, the concept of “unawareness” is also not without conceptual issues. In the context of the procedure we use here, we consider it sufficient to describe an assessed behavior as relatively unaware when individuals do not need to directly reflect upon the belief under investigation to provide a behavioral response (in contrast to self-report measures where such reflection is required).

“Traditional” implicit measures, like the IAT, are not without problems and limitations (for summaries of recent controversies, see^20–24^). These association-activation approaches, are rooted in semantic network models^25^. They posit the idea that the activation of a mental concept in memory (e.g., the word “I”) automatically activates other associated concepts (e.g., “amazing”)^26^. Performance-based implicit measures are supposed to capture the strength of this association (e.g., via response times^23^). Such association-activation approaches have historically dominated the field of implicit measures. Recently, however, some authors have argued their limitation since they do not specify relations between associated concepts [e.g.,^27–29^] For instance, a belief such as “intelligence is changeable” is different (and would relate to behavior differently) compared to a belief such as “intelligence should be changeable”.

In response to these limitations, a new range of implicit measures known as *relational implicit measures* have been developed [e.g.,^30^]. These measures are specifically designed to capture relational information between concepts, and have already provided novel insights in other areas of psychology [e.g.,^12^] and groundbreaking explanations for some contradictory findings in research on evaluations [e.g.,^31^]. For instance, an early study found that depressed individuals show more positive evaluation of the self (on traditional implicit measures), compared to non-depressed individuals^32^. This finding was somewhat counterintuitive. However, Remue et al.^33^ assessed implicit self-esteem in participants who had a low or high tendency for depression using a relational implicit measure, which enabled the assessment of two separate beliefs: “actual” self-esteem (“I am good”) and “ideal self-esteem” (“I want to be good”). Participants with a high tendency for depression scored higher for ideal self-esteem and lower for actual self-esteem, indicating that a *discrepancy* between these beliefs appears to characterize the responses of highly depressed individuals. Such an insight would not be possible using traditional implicit measures. By leveraging relational implicit measures as a methodological tool, we can validate and delve deeper into the theoretical intricacies of implicit beliefs about intelligence.

In our studies, we chose to adapt one of the most promising relational implicit measures, the Propositional Evaluation Paradigm (PEP^34,35^), to assess intelligence mindsets. The PEP is a sequential priming task that presents statements from existing questionnaires on the screen. First, a prime statement (e.g., “Everyone has a certain level of intelligence that can be changed”) appears in the middle of the screen in a word-by-word fashion. Then, during prompt trials a required response prompt appears (TRUE or FALSE) and participants need to push a button (in the reaction-time version) or move their mouse (in the mousetracking version) to true or false in the upper part of the screen. Filler (“catch”) trials are integrated in the measure to assure that people read the statements: in one variant they need to judge whether the statement was correctly spelled (spelling-error variant), in another variant they need to evaluate the statements’ truth value (truth-evaluation variant). If a person’s belief is congruent with the prime statement, their mouse-movement (in the mousetracking version) would be more direct towards the response “true” at the prompt trials or they would be quicker at pushing the “true” response key (in the reaction time version) as compared to a person that does not share a presented belief. In seminal articles of the PEP^34,35^, anti-immigrant beliefs at the individual level assessed with the measure successfully predicted explicit anti-immigrant beliefs and willingness to exert effort in order to donate to a charity working against discrimination.

## Present research

Across two studies, we aimed to test whether stronger growth mindsets assessed using a relational implicit measure (the mousetracking-based PEP^34^) predicted higher post-failure learning behavior in an IQ assessment situation. Mousetracking is a superior method at capturing the nuances of evaluation dynamics [e.g.,^36^] and it has been recently found to be more sensitive to relational information than the reaction time version^37^. In both studies, participants were invited for an IQ assessment to the laboratory where they first completed the PEP adapted to assess intelligence mindset. Then, all participants attempted to solve a block of very difficult IQ test items [e.g.,^38^]. Subsequently, participants received performance feedback (which was low overall, creating a failure experience, which is a crucial theoretical condition for mindsets to become relevant [e.g.,^6^]. Thereafter, they had the opportunity to learn how to solve the difficult IQ items. Time spent on learning about the solutions (time-based learning) and the number of solutions reviewed (item-based learning) served as behavioral indicators for engagement in learning^39^.

In both studies, our preregistered confirmatory hypothesis was that the PEP measure of growth mindset would predict post-failure learning behavior. We measured various learning behaviors in both studies—choice to review solutions, number of solutions reviewed, and time spent on reviewing the solutions—which can serve as different indicators of learning engagement. The only preregistered control variable was self-efficacy because it is known to affect motivation in achievement situations^40,41^ and has been used in similar studies on mindset, exploring the effect of growth mindset in the face of setbacks [e.g.,^42^].

### Study 1

In Study 1 (*N* = 184), we assessed implicit growth mindset with the spelling-error variant of the mousetracking PEP^34^.

#### Results

There are two analyses presented in the Results section of Study 1. An exploratory analysis complements the pre-registered linear regression by addressing the data's distributional characteristics, which we did not expect at the time of pre-registration. The first step of the two-step models account for the zero-inflated nature of the outcomes, providing a more nuanced understanding of the underlying processes influencing these learning behaviors (see further details in the Statistical Analysis section).

##### Linear regression (preregistered analysis)

The preregistered analysis showed that a stronger implicit growth mindset was associated with more time spent on post-failure learning (*b* = 22.03, 95% *CI* [2.19, 41.88], *t*(181) = 2.19, *p* = 0.030), while controlling for self-efficacy (*b* = 25.30, 95% *CI* [5.45, 45.15],* t*(181) = 2.515, *p* = 0.013). Furthermore, we analyzed our secondary pre-registered dependent variable (i.e., the number of solutions reviewed). Similarly, we found that a stronger implicit growth mindset was associated with an increased number of items reviewed (*b* = 0.61, 95% *CI* [0.10, 1.13], *t*(181) = 2.36, *p* = 0.019), while controlling for self-efficacy (*b* = 0.86, 95% *CI* [0.35, 1.38], *t*(181) = 3.31, *p* = 0.001). Thus, our preregistered hypothesis was fully supported by both preregistered dependent variables, a stronger implicit growth mindset was associated with higher engagement in learning.

##### Two-step model (exploratory analysis)

The normality assumption of residuals of the pre-registered analyses were not met (as assessed by a Kolmogorov–Smirnov test; *D* = 0.167, *p* < 0.001). Therefore, first, we transformed the primary outcome variable and applied a two-step model (logistic and linear regressions), where we were interested in the linear part of the model (see explanation in the statistical analysis section). We found that a stronger implicit growth mindset was not predictive of the choice to view any solutions in the logistic model (*OR* = 1.13, 95% *CI* [0.70, 1.87], *p* = 0.635), when controlling for self-efficacy (*OR* = 1.46, 95% *CI* [0.92, 2.33], *p* = 0.107). However, implicit growth mindset predicted the time spent viewing the solutions among those who chose to view any solutions in the linear model, when controlling for self-efficacy (see Table 1). Thus, those with stronger implicit growth mindsets dedicated a greater amount of time to learn from their mistakes.

**Table 1:** The relationship between implicit growth mindset and time-based and item-based learning behaviors in both studies, when controlling for self-efficacy.

	Time-based learning						Item-based learning				
	Predictors	Estimates	SE	CI	Statistic	*p*	Incidence rate ratios	SE	CI	Statistic	*p*
Study 1 (*N* = 164)	(Intercept)	10.71	0.40	9.91–11.51	26.49	** < 0.001**	5.17	0.17	4.85–5.51	50.95	** < 0.001**
	Self-efficacy	0.94	0.41	0.14–1.75	2.31	**0.022**	1.17	0.04	1.09–1.25	4.70	** < 0.001**
	Implicit growth mindset	0.83	0.41	0.02–1.64	2.03	**0.044**	1.14	0.04	1.07–1.21	4.16	** < 0.001**
Study 2 (N = 177)	(Intercept)	12.17	0.44	11.30–13.04	27.47	** < 0.001**	6.29	0.17	5.96–6.64	66.91	** < 0.001**
	Self-efficacy	0.45	0.44	− 0.42–1.33	1.02	0.308	1.07	0.03	1.01–1.13	2.41	**0.016**
	Implicit growth mindset	0.89	0.44	0.01–1.77	2.00	**0.047**	1.14	0.03	1.08–1.20	4.72	** < 0.001**

The table represents the second step of the two-step models (i.e., the analysis of our interest)—see explanation under Statistical Analysis section. Significant values are in bold.

For the secondary outcome, we applied a right-censored Poisson regression (see details in the statistical analysis section), which supported the main analysis: we found that implicit growth mindset also predicted the number of solutions reviewed among those who chose to view any solutions, when controlling for self-efficacy (Table 1). Those individuals who had a stronger implicit growth mindset reviewed more solutions.

##### The role of explicit growth mindset (exploratory analysis)

Our aim with these analyses was to explore if implicit growth mindset explained any variance in addition to explicit growth mindset for which we further applied the two-step model. We found that people endorsing a stronger implicit growth mindset spent significantly more time on viewing the solutions (*b* = 0.86, 95% *CI* [0.06, 1.67], *p* = 0.035), while explicit growth mindset was not a significant predictor (*b* = 0.73, 95% *CI* [-0.06, 1.53], *p* = 0.071), when controlling for self-efficacy (*b* = 0.90, 95% *CI* [0. 10, 1.71], *p* = 0.027). In the binomial part of the two-step model, none of the variables predicted the decision to view solutions (implicit growth mindset: *OR* = 1.13, 95% *CI* [0.70, 1.89], *p* = 0.624; explicit growth mindset: *OR* = 1.20, 95% *CI* [0.76, 1.88], *p* = 0.419; self-efficacy: *OR* = 1.45, 95% *CI* [0.91, 2.32], *p* = 0.116).

**Figure 1:**
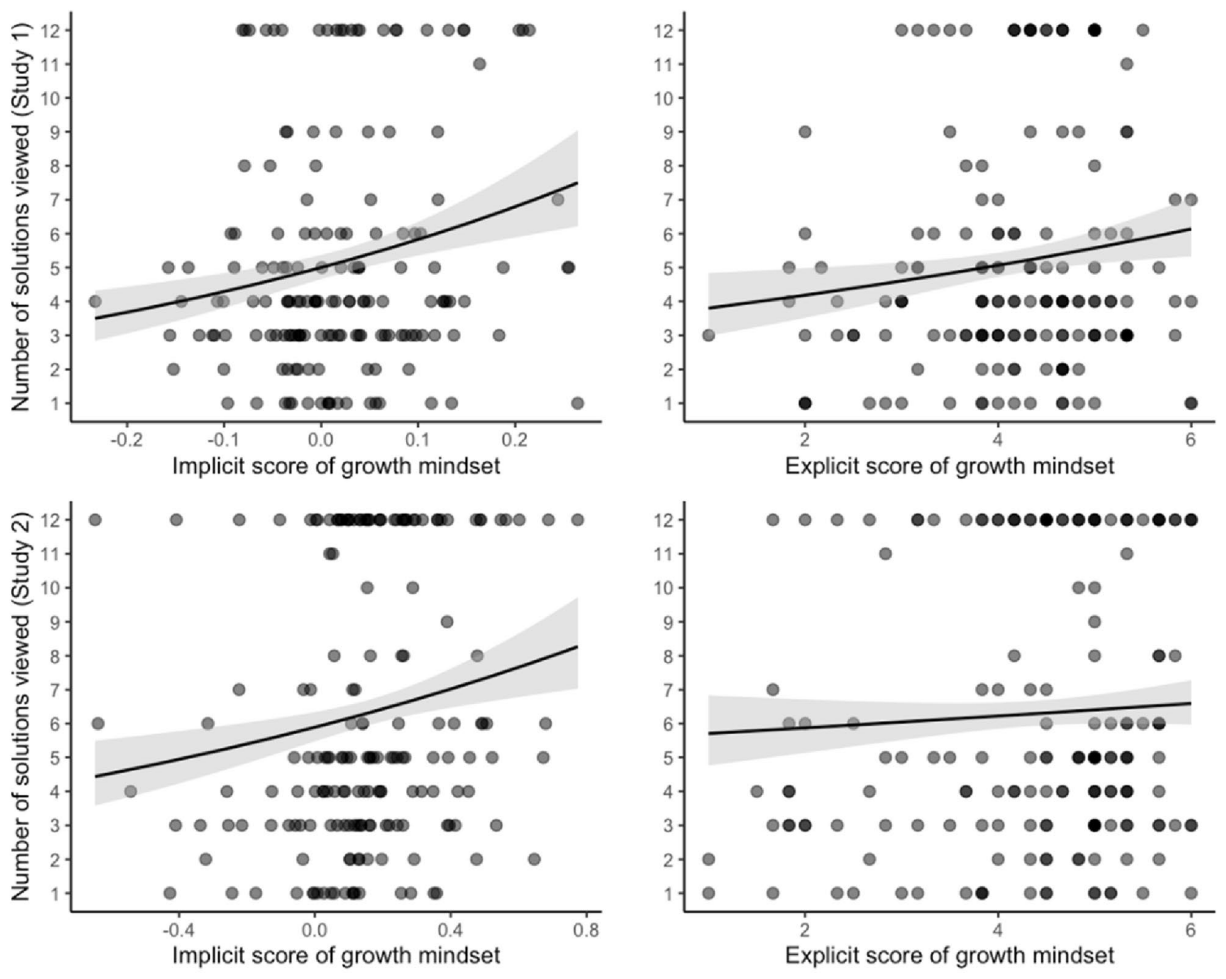
Results from the right-censored Poisson model. *Notes.* The figure shows the association between the implicit and explicit scores of growth mindset and item-based learning, when controlling for self-efficacy. The grey area shows confidence intervals of predicted values. Study 1: *N* = 164; Study 2: *N* = 177.

Moreover, we applied the same censored Poisson model among the nonzero values to predict the secondary dependent variable, including the explicit score of intelligence mindset. Here we found that both the implicit (*IRR* = 1.14, 95% *CI* [1.08, 1.22], *p* < 0.001) and explicit scores of growth mindset (*IRR* = 1.10, 95% *CI* [1.03, 1.18], *p* = 0.007) predicted learning behavior (i.e., the number of items viewed), when controlling for self-efficacy (*IRR* = 1.16, 95% *CI* [1.09, 1.24], *p* < 0.001). The relationship is represented in Fig. 1.

### Study 2

The aim of Study 2 (*N* = 193) was to replicate the results of Study 1 with an improved and more reliable measure assessing intelligence mindsets implicitly. Specifically, we used the truth-evaluation variant of the PEP, which had previously demonstrated higher reliabilities than the spelling-error variant^34,35^ and made further adjustments to make the task more reliable and user-friendly. Otherwise, the procedure and design of the study was the same as in Study 1.

#### Results

##### Two-step model (preregistered analysis)

Based on the data of Study 1, we expected that the number of viewed solutions will not be normally distributed. Therefore, in Study 2, we directly pre-registered the two-step model. Consistent with our preregistration, when holding self-efficacy constant (*OR* = 0.99, 95% *CI* [0.59, 1.67], *p* = 0.984), the decision to view any solutions was not associated with a stronger implicit growth mindset (*OR* = 1.10, 95% *CI* [0.65, 1.80], *p* = 0.717). However, among those who decided to view at least one solution, higher implicit growth mindset predicted higher engagement in learning in terms of time spent on looking at the solutions, while controlling for self-efficacy (Table 1). Furthermore, replicating the results of Study 1, a higher implicit growth mindset was associated with increased number of solutions in the right-censored Poisson regression among those who decided to view at least one solution, when controlling for self-efficacy (Table 1). Thus, again, our preregistered hypotheses was fully supported.

##### The role of explicit growth mindset (exploratory analysis)

As expected, the decision to view any solutions was not predicted by the implicit (*OR* = 0.97, 95% *CI* [0.45, 2.08], *p* = 0.947) or explicit (*OR* = 1.17, 95% *CI* [0.55, 2.50], *p* = 0.680) growth mindsets, when controlling for self-efficacy (*OR* = 1.01, 95% *CI* [0.60, 1.70], *p* = 0.972). Furthermore, contrary to the findings of Study 1, neither implicit (*b* = 0.85, 95% *CI* [-0.46, 2.15], *p* = 0.201) nor explicit growth mindset (*b* = 0.06, 95% *CI* [-1.25, 1.36], *p* = 0.934) predicted the time-based learning variable, when controlling for self-efficacy (*b* = 0.46, 95% *CI* [-0.43, 1.35], *p* = 0.308). However, people endorsing a stronger implicit growth mindset (*IRR* = 1.11, 95% *CI* [1.03, 1.20], *p* = 0.009) reviewed an increased number of solutions as shown by the right-censored Poisson regression among those who reviewed at least one solution, even after controlling for explicit growth mindset (*IRR* = 1.04, 95% *CI* [0.95, 1.13], *p* = 0.414) and self-efficacy (*IRR* = 1.07, 95% *CI* [1.01, 1.13], *p* = 0.013). Contrary to our findings in Study 1, explicit growth mindset was predictive (IRR = 1.12, *p* < 0.001) only if we did not include implicit growth mindset in the model—once we included implicit growth mindset, the explicit effect was no longer significant. The relationship of implicit and explicit growth mindset is represented in Fig. 1.

## Discussion

Bridging implicit measures and growth mindset theory together, this research represents the first exploration into the implicit theories of intelligence mindset beyond self-report. Two studies confirmed our preregistered hypothesis: individuals with a stronger implicit growth mindset showed higher engagement in a learning task after failure. Moreover, their behavior was predicted by implicit growth mindset above and beyond the traditional self-report measure. Thus, our research adds a new layer of nuance to research on growth mindset by suggesting that implicit measures may provide insights into this phenomenon which are not fully captured by explicit measures. However, it is important to note that the use of these different measures does not imply that separate constructs are being assessed. In line with recent theorizing^27,31^, we would suggest that the construct captured within these measures is likely identical; what differs between measures is simply the conditions under which the construct is assessed^43^. Between the PEP and self-report of growth mindset, the conditions which vary relate to both awareness of the influence of beliefs on responding and the required speed of responding to these beliefs.

One point which emerges with regard to measurement specifically relates to the intertwined nature of “unawareness” and “fast” responding. These conditions were significantly influenced by the design of catch trials in the studies. Catch trials, which are unique trials integrated in the measure to ensure participant engagement, differed significantly between our two studies. In Study 1, these trials focused on spelling accuracy and did not require participants to engage with their beliefs about the statements, leading to more automatic, "unaware" responses across all trials (including probe trials, which were used to measure participants’ beliefs). This was reflected by the lack of correlation between explicit and implicit measures. In contrast, Study 2’s catch trials required participants to actively agree or disagree with the statements, thus directly evoking their explicit beliefs. This direct engagement with the content resulted in the implicit and explicit measures being strongly correlated. Given these correlational patterns and the irrelevance of beliefs to the entirety of the measure in Study 1, it could well be argued that Study 1’s PEP involved rather more “unaware” responding than in Study 2. However, this unawareness was coupled with poor measurement properties, which would limit the utility of the measure in predicting reliable individual differences. Indeed, a similar confound of reliability and (un)awareness has been noted in other studies^34^. In any case, decoupling the relative importance of “unaware” vs. “fast” conditions of responding in maximizing the usefulness of measures of growth mindset represents an important next step for this research agenda to address.

Our methodological approach of interfacing research on intelligence mindset with implicit social cognition may provide new perspectives on existing research findings. Dweck^44^ described people who report a stronger growth mindset but do not behave accordingly as possessing a “false growth mindset”. Our approach and results tell a different story: it may be that these individuals exhibit a stronger growth mindset in self-reports, but this may not persist when assessed using implicit measures. When given time to reflect and deliberate, parents and teachers who have learned about the theory might explicitly embrace a stronger growth mindset (particularly given the widespread knowledge of the concept in contemporary educational contexts). However, in more spontaneous situations, such as when reacting to children’s success, other beliefs may come to the fore. For example, teachers might quickly react to a child’s success by saying the well-known “you’re so smart” and only after some time realize they should have said “great work”—praising the process instead of the person^45^.

As argued in the introduction, bridging growth mindset theory and implicit measures may also provide some context and explanation for the issues relating to replicability which are present in the growth mindset literature^13–15^. For instance, most of these studies used complex achievement outcomes (e.g., academic grades) while assessing intelligence mindsets using only self-reports. Academic grades result from students’ educational achievement throughout a whole semester or a term, potentially reflecting different types of behaviors—behaviors where deliberation is required (e.g., making study plans) but also behaviours which are emitted more quickly (e.g., one’s immediate response to a challenge issued in class). For such outcomes, multimodal approaches to measurement would be advantageous to reflect the full continuum of these processes.

Future research could provide empirical evidence for specific conditions where the application of implicit versus explicit measures of growth mindset would be superior in predicting behavior. We suggest that explicit measures may be particularly valuable in settings where individuals have the time and resources to reflect on their beliefs, such as in making study plans. In contrast, implicit measures may be more useful in contexts where responses are spontaneous, such as real-life classroom interactions, where students' immediate responses to challenges may be guided by their implicit beliefs. Sometimes, for instance regarding educational grades which reflect the combination of those spontaneous and more reflective behaviors, a multimodal approach could be employed utilizing both implicit and explicit measures.

Our approach here also creates opportunities to explore new theoretical ideas. For instance, some research on implicit-explicit discrepancies has suggested that it is relatively easy to change explicit attitudes, but implicit attitudes are deeply rooted and quite rigid to change [e.g.,^46–48^]. If growth mindset beliefs can be viewed both explicitly and implicitly, they may also be subject to these phenomena, and theoretical frameworks should evolve to account for the pontential impact of both. An extensive amount of research has documented that people’s explicit growth mindset can be promoted by various forms of persuasive messages^3,5,49–51^. In everyday contexts such growth messages were in the last decades vastly conveyed by the media^52,53^, in bestseller books [e.g.,^54^], and in education and corporate cultures (e.g., Microsoft^55^). If such explicit beliefs are frequently retrieved, they may become more automatized over time and guide behavior even under suboptimal conditions [e.g.,^56,57^]. Future research could test the effectiveness of intense, repeated growth mindset interventions at the implicit level, as it is possible that such interventions may have the greatest impact when they also affect implicit scores. We assume that any intervention will require participants to access the growth belief repeatedly including situations that involve quick and relatively unaware responding (e.g., when reacting to failure) to foster the development of an automatic growth-oriented response.

We present standardized effect sizes of Pearson's r in Table 2, which shows that the effect sizes between implicitly measured growth mindset and post-failure learning behaviors are modest (ranging between 0.14 and 0.18). This correlation is statistically considered small, however it is practically significant within the context of educational psychology. As Hill et al.^58^ suggest, effect sizes in educational research should be considered in relation to field-specific benchmarks and their practical implications rather than merely their statistical significance. Regarding educational learning behaviors, the cumulative effect that may occur over time can be substantial. Repeatedly engaging in learning behaviors can lead to significant changes in more holistic learning outcomes, such as real-life achievement scores. The correlation we observed highlights the modest yet potentially impactful relationship between a student’s implicit growth mindset and their behavior following failure.

**Table 2:** Zero-order correlations, descriptive statistics and reliabilities of variables of interest.

Variable	Study	*M*	*SD*	Reliabilities (*Ω* or split-half*)*	1	2	3	4	5
1. Time-based learning	1	10.71	5.28	–	–	.83** [.78, .87]	.15* [.00, .29]	.11 [− .04, .25]	.08 [− .07, .22]
	2	12.17	5.95	–					
2. Item-based learning	1	5.16	3.44	–	.84** [.78, .88]	–	.18* [.03, .32]	.15 [− .00, .29]	.09 [− .06, .24]
	2	6.13	3.97	–					
3. Implicit growth mindset	1	0.02	0.09	− 0.08	.14 [− .02, .28]	.16* [.01, .31]	–	.74** [.66, .80]	.00 [− .14, .15]
	2	0.15	0.24	0.94					
4. Explicit growth mindset	1	4.17	0.98	0.94	.14 [− .01, .29]	.13 [− .02, .28]	− .05 [− .20, .10]	–	− .08 [− .23, .07]
	2	4.36	1.20	0.97					
5. Self-efficacy	1	3.59	0.68	0.89	.16* [.01, .30]	.19* [.04, .34]	− .13 [− .27, .03]	.06 [− .10, .21]	–
	2	3.63	0.64	0.86					

*M* and *SD* are used to represent mean and standard deviation, respectively. Values in square brackets indicate the 95% confidence interval for each correlation. Correlations of Study 1 are presented in the bottom left part of the table and correlations of Study 2 are presented in the upper right part of the table. Analyses were run among participants who reviewed at least one solution—see explanation under Statistical Analysis section (Study 1: *N* = 164; Study 2: *N* = 177).

Although the majority of this paper has focused on the ways in which advances from implicit cognition research can improve research on intelligence mindset, it is worth noting that our results here also have implications for theoretical accounts on implicit cognition. Critically, our results add yet further evidence to the growing body of work that suggests that relational information plays a critical role in effects on implicit measures [e.g.,^31^]. Indeed, relational implicit measures^35,59^, have opened further new doors for exploration with these measures and the necessity to consider implicit beliefs (rather than merely implicit attitudes). At the mental level, our results provide support for the propositional perspective of implicit evaluation: namely, that complex propositional belief-structures are captured at the automatic level by implicit measures^60^. Despite its potential advantages (e.g., in terms of more precise access to the construct measured^34,59^), this propositional approach is still less widely-considered than the associative approach. Most critiques of implicit measures have targeted the associative approach (e.g., the Implicit Association Test^61^). Our study suggests that beliefs reflecting relational information captured at the implicit level provide predictive utility beyond their explicit counterpart, as well as very high reliabilities. Thus, future studies could apply these novel relational implicit measures in other areas of implicit social cognition to explore whether the historical promises of implicit measures (e.g., predicting behavior above and beyond explicit measures^17,43^) might be better substantiated by this novel approach.

Our research opens a new door to explore the multifaceted nature of "implicit theories", nevertheless, there are several key limitations that must be acknowledged. First, as already mentioned, the entangled nature of fast vs. unaware responses should be dealt with in future research. However, there is a further issue of entanglement present in our work: the entanglement between “varying conditions” and “measurement error”. As Schimmack^24^ and others have noted, in many situations implicit measures represent the more noisy measurement of the same construct captured by their corresponding self-report equivalent. Although we do not suggest that the construct assessed in our implicit measure differed from the self-report, we do assume that divergence observed between the measures was attributable to different response conditions (e.g., faster and less aware), rather than due to differences in measurement precision. For instance, our earlier interpretation that Study 1’s measure represented a “more unaware” measure than in Study 2 due to the relatively lower correlation with the explicit measure could also be interpreted as the measure in Study 1 simply being noiser than in Study 2 (and indeed, this is supported by the much higher reliability of the measure in Study 2 compared to Study 1). Given the extensive contemporary theoretical and conceptual challenges present in implicit measures research, the onus is on further research to demonstate more definitively that variations in these features are meaningful beyond differences in measurement error. This aside, one further issue with our study is more practical: while the implicit measure holds promise, when contemplating its adaptation for research studies, it is important to recognize that its completion time is significantly longer compared to its explicit counterpart. We attempted to adopt a shorter version of the measure in Study 1, however its internal reliability was very poor, therefore we needed to increase the trial numbers in Study 2. This limitation should be addressed in the future, especially if one desires to use the measure in shorter studies.

In sum, our results suggest that bridging measurement and theory in intelligence mindset research represents a useful future avenue for work, which may help to shed light on puzzling patterns of results present in the literature. It is essential to recognize that while our study introduces a nuanced perspective by incorporating implicit measures into the assessment of intelligence mindsets, we do not claim to invalidate established accounts. Rather, we seek to enrich them. Existing explicit measures and the implicit measure we employed here provide complementary insights, highlighting the importance of considering both deliberative and more automatic processes to more wholly investigate growth mindsets in the future.

## Methods

Both studies were approved by the institutional review board of the Department of Occupational, Economic and Social Psychology at the University of Vienna. Furthermore, both studies were conducted in accordance with the Declaration of Helsinki and we obtained informed consent from all participants.

### Study 1

#### Participants

We recruited a sample via the psychology student credit pool at the University of Vienna. Participants received partial course credits for taking part in our study. A Monte Carlo power analysis (details can be found in the preregistration) determined that we need 155 participants for this study. We preregistered to recruit 220 participants, and upon reaching 220 participants finishing the protocol, we stopped data collection. After applying the preregistered exclusion criteria (2 were non-native German-speaking, 5 wished not to include their data, 29 achieved less than 80% accuracy (see measures section) on the implicit measure), we included 184 students’ (mean age = 21.32, *SD* = 4; 76% female) data.

#### Procedure

We collected data in a lab at the university. Participants were informed that we would like to better understand how students integrate information and how they differ in their intellectual abilities. The research protocol was fully computerized on Qualtrics and lab.js. After participants consented to the study and approved the data protection form, during the assessment phase I, they completed the intelligence mindset PEP, a measure of self-efficacy, and some other questionnaires including the explicit intelligence mindset scale. Next, they entered the failure experience phase where they worked on a series of 12 mostly very difficult IQ problems. Participants received performance feedback, which was very low overall (*M* = 2.78, *SD* = 1.61). Subsequently, in assessment phase II, they could voluntarily look at the solutions for each IQ-problem they had worked on before. The time spent in this phase as well as the number of problems looked at served as the dependent variables in the study. To buffer possible negative emotional effects of the failure experience, we included a final success experience block. Participants worked on a series of easy and medium difficulty IQ problems without a time limit. Finally, participants responded to demographic questions, and were thanked and debriefed.

#### Measures

The pre-registration includes all measures we assessed for exploratory purposes; however here we only report variables relevant to our research question. A complete list of included measures is presented in the supplement (S1). Descriptive statistics, zero-order correlations and reliabilities are presented in Table 2.

##### Implicit measure of growth mindset

Cummins and De Houwer^34^ developed the mousetracking PEP used in this study on lab.js^62^ to assess anti-immigrant beliefs. We adapted the program to assess intelligence mindsets and embedded the measure in Qualtrics. In the measure, participants were presented with items from the German version of the theories of intelligence scale^63^ in a word-by-word fashion. This version of the scale originally consisted of 3 items, presenting a growth and a fixed option at the ends of a Likert scale (e.g., Everyone has a certain level of intelligence that (1) “*cannot be changed*”—(5) “*can be changed*”). Guided by the best practices established by Müller & Rothermund^35^, we opted for clarity by distinctly presenting both positively and negatively phrased items. Hence, participants were introduced to standalone statements like “Everyone has a certain level of intelligence that can be changed”, resulting in a total of 6 item statements.

**Figure 2:**
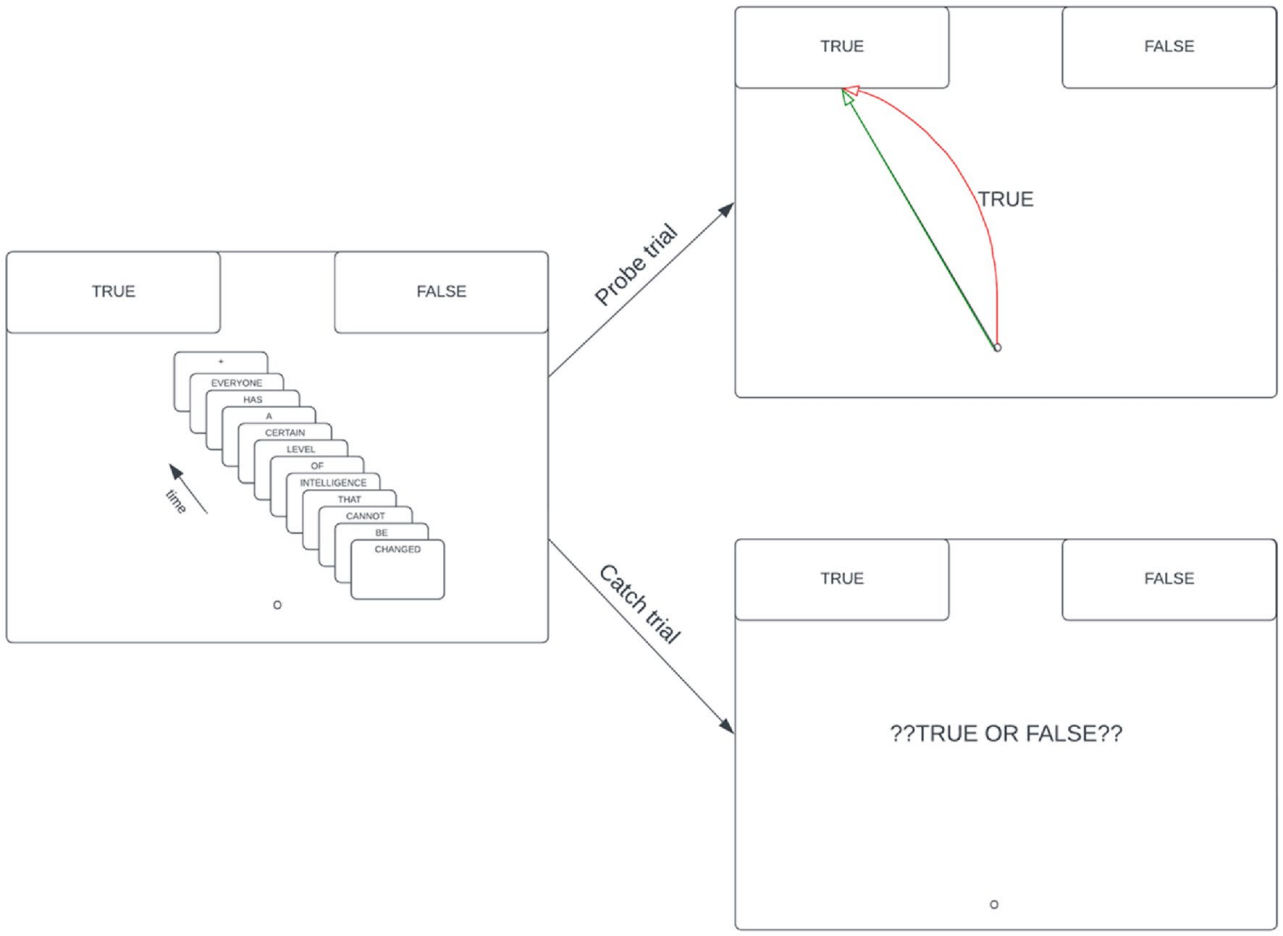
Example Item of a Probe Trial in the PEP. Notes. The figure represents a trial from the measure followed by a probe or catch prompt. The prime statement is translated and adapted to English (the study used the German items). The words were presented in the middle of the screen, one by one. Time limits for words in Study 1 were determined by the recommendation of the original PEP measure^35^, meaning that every word had a base time limit (150 ms) and with every letter, the time limit increased by 25 ms. Time limits for words in Study 2 were defined following learnings from the mouse-tracking PEP studies^34^, thus each word was presented for 200 ms. Participants needed to respond to the probe or catch prompts in under 2000 ms. The image in the upper right corner shows that the mouse movement deviation from the neutral or “optimal” path (black) towards true (green) or false (red) creates an area under the curve in each trial. The final implicit score was drawn from participants’ time-normalized average trajectories across all trials. For instance, someone with a strong growth mindset would be represented by the green trajectory in the image.

Upon statement presentation, participants engaged with subsequent “TRUE” or “FALSE” prompts (as detailed in Fig. 2) via moving their mouse from a bottom-center starting point, leading either to the top-left ("true") or top-right ("false") screen corners. The task was to respond according to the prompt (e.g., selecting "false" when presented with "FALSE"). We integrated so called *catch trials,* a technique designed to ensure sincere engagement with the statements^34,35^. These catch trials appear at random time points within the task and are identifiable by a distinct prompt (“??TRUE/FALSE??”). When this prompt appears participants have to decide whether the statement was spelled correctly (“*true*”) or incorrectly (“*false*”). Because the prompts appear after the statements participants don’t know whether a trial will be a regular trial or a catch trial. Accordingly, they have to read every statement carefully.

Both the correctly and incorrectly spelled versions were presented twice. They were followed twice by two types of probe trials (i.e., with the prompt “*TRUE*” twice, and the prompt “*FALSE*” twice). Furthermore, both the correctly and incorrectly spelled versions of the statements were followed by a catch trial (i.e., the prompt “??*TRUE/FALSE*??”) twice. Thus, the PEP consisted of 72 trials in total (6 statements × 2 spelling versions × 2 “*TRUE*” prompt × 2 “*FALSE*” prompt + 6 statements × 2 spelling versions × 2 catch trials). Accuracy on the measure reflects the ratio of correctly responding to “*TRUE*” and ”*FALSE*” prompts and correctly spotting the spelling errors on catch trials.

We registered the area under curve (AUC^64^) of participants’ responses and calculated participants’ time-normalized average trajectories across all trials. Greater deviation from the optimal trajectory indicates a smaller automatic tendency to agree (“*true*”) or disagree (“*false*”) with the presented item. We created the implicit score of intelligence mindset, using the method and code of Cummins and De Houwer’s^34^ experiments. The implicit score was coded in a way that higher scores represent more of a growth mindset.

##### Explicit assessment of intelligence mindsets

We assessed self-report intelligence mindsets with the items used in the PEP^63^. Participants responded to 6 items on a Likert scale from 1 (*strongly disagree*) to 6 (*strongly agree*). Three items from the scale were reverse coded and the score was aggregated in a way that higher scores represent a stronger growth mindset belief.

##### Self-efficacy regarding the IQ tasks

Self-efficacy was measured via 7 statements (e.g., “I believe I will succeed in this IQ task”) adapted from the self-efficacy subscale of the Motivated Strategies for Learning Questionnaire (MSLQ^65^). Participants responded on a Likert scale from 1 (*strongly disagree*) to 6 (*strongly agree*). The score was aggregated in a way that higher scores represent higher efficacy regarding the IQ tasks.

##### Failure experience

Participants completed a block of 12 items (1 easy, 1 medium, 10 difficult) from the Raven’s Progressive Matrices [e.g.,^66^] with 45 s time limit per item^38^. We included 1 easy and 1 medium difficulty item at the beginning of the block, in order to ensure that participants had the feeling that some items were possible to solve. Subsequently, they received performance feedback, which displayed (in red text) that they achieved X/12 scores. On this relatively difficult block, we aimed to induce a failure experience. On average, participants completed 2.8 items correctly. We additionally assessed participants’ perceived failure experience on this block with 1 item (“How well did you do on the warm-up exercises?”). Participants responded on a scale ranging from 1 (*not good at all*) to 6 (*very good*). We found that 69% of the participants chose not good at all, 21% said predominantly not good, 9% said rather not good, 1% said rather good and no one chose predominantly good or very good. This means that most participants experienced failure on this block.

##### Post-failure learning behavior

Participants could review the solutions of items they worked on in the difficult warm-up block. First, they could choose whether they wished to see any solutions or rather proceed to the “main IQ assessment” (we communicated to participants that the subsequent block was the main IQ assessment; however, we were not interested in performance on that block). Subsequently, if they decided to view the solutions, we showed them an explanation of the first item they worked on. These explanations were similar to Porter et al.’s^39^ effort variable in the PERC (persistence, effort, resilience and challenge-seeking) task, which was developed to assess growth mindset mastery behaviors. The explanations included the response they gave, the correct response, and then the picture of the IQ task. The solution of the IQ task was then explained by a text (between 30–40 words) beneath, including two to three bullet points, followed by a closer screenshot to visualize the bullet points.

We pre-registered two dependent variables regarding post-failure learning. First, we administered the time spent on viewing the solutions. For our main analysis we created a learning index of the time spent on this block in a way that higher scores represent greater amount of learning behavior. Second, after each solution, students could decide if they wished to view the next item’s solution or proceed to the “main IQ assessment”. Thus, our secondary dependent variable was the number of solutions participants decided to view. This number ranged from 0–12, with higher scores reflecting more learning behavior. The high correlation between the two measures (Table 2), reflects a strong but not perfect relationship, suggesting that they might carry some distinct aspects of learning engagement but basically converge into one construct.

##### Success experience

In the last part of the study, participants were presented with 12 Raven items (including 4 easy, 4 medium difficulty and 4 difficult IQ items) with no time limit. The aim of this block was to create a failure-buffering success experience for participants (mean performance score was 10.1/12—this was the case because participants could spend as much time as they needed to solve the items on this block). Thus, this block was not in the focus of our study.

#### Statistical analysis

In the results section, first we introduce the pre-registered analysis with linear regression on the two learning scores (time-based and item-based), predicted by the implicit score when controlling for self-efficacy. Next, we provide exploratory analyses: first, testing the hypothesis with outcome-adjusted models (i.e., two-step models); second, including the explicit scale in multiple regressions; third, conducting hierarchical linear regressions to test the additive standardized effects of the implicit and explicit scores.

In the pre-registered analysis, the primary outcome variable showed a heavily right-skewed distribution, bounded at zero (11%). To account for the right-skewed distribution, we can apply a square-root transformation to create a more normally distributed variable^39^. However, this transformation will not account for the zero values in the data. For outcome variables bounded at zero, we need to apply a two-step model, because in reality, two processes occur: one that governs the choice of viewing any of the solutions, and one that determines the time an individual spends on the solutions if they decide to view any of them (e.g.,^67^). For the first part of the model, we can apply a logistic regression with a binary outcome variable to predict zero and nonzero values. For the second part of the model, we can apply a linear regression predicting the transformed non-zero values. This is an important differentiation, because the decision to view any solutions might be motivated by other reasons (e.g., wanting to finish the study earlier). Thus, the main interest of our analyses is the linear regression part of the model.

Next, we tested our hypothesis with a more appropriate model on the secondary preregistered dependent variable, i.e., the number of solutions participants optionally viewed (item-based learning). Even though applying Poisson regressions is the go-to method to analyze count data, in our model the assumptions for Poisson regression were not met. 11% of participants chose not to view any solutions (zero-inflation) and 12% of participants viewed all solutions (resulting in a right censored outcome variable). Thus, we followed a similar procedure to the previous analysis, using a two-step model^68^. First, a binomial regression is necessary to predict zero and non-zero values. Next, including only the non-zero values, we need to account for the right censored nature of the data with a right-censored Poisson regression. As the binomial regression would show the same results as in the previous analysis (binomial model part), we only report the results of the censored Poisson regression.

### Study 2

#### Participants

We recruited a sample via the psychology student credit pool at the University of Vienna. Participants received partial course credits for taking part in our study. An analysis in G*Power (details can be found in the preregistration) determined that we need 160 participants for our main analysis in the linear model (including only those who reviewed at least 1 solution). We recruited 208 participants to account for the exclusion criteria. After applying the exclusion criteria (3 did not finish the protocol, 3 were non-native German-speaking, 4 wished not to include their data, 5 achieved less than 80% accuracy on the implicit measure—see measures section), we included 193 students’ (mean age = 21.6, *SD* = 3.73; 73% female) data—and 177 students reviewed at least one solution.

#### Procedure and measures

The procedure, design and most measures (except for the implicit measure) were the same as in Study 1.

#### Implicit measure of growth mindset

We adapted the truth-evaluation variant of the PEP measure for this study^34^. We made four changes in this measure compared to the one we used in Study 1. First, we integrated additional practice trials (3 × 10 trials of each type), to ensure that participants better understood the task, aiming to reduce attrition rate. Second, to make sure that participants read the prime statements, on catch trials (??*TRUE/FALSE*??), they agreed or disagreed with the statements by moving the mouse to true or false at the top of the page (instead of spotting a spelling error on catch trials). Third, we included more trials in this measure to increase reliability. We determined the number of trials based on an online pilot study (N = 18), where we included 344 trials per participant to predict the reliability estimate with different trial number increments. Our analyses proposed that if we included ~ 140 prompt trials (we included 144), the reliability estimate would be Rsb = 0.84 (95% CI [0.57; 0.94]). As we ran our study in the lab, we expected a higher reliability estimate than the predicted one, and due to the larger sample size, we expected the confidence intervals to be smaller. Fourth, as we included more trials, we reduced the time interval of each word that was presented on the screen to keep the time spent on this measure bearable—instead of having a base time limit (150 ms) and adding 25 ms with each letter, all words were presented for 200 ms (which was shown to be successful in Cummins & De Houwer’s^34^ experiments). 72 statements were followed by the prompt TRUE (36 statements were positively and 36 statements were negatively phrased). Another 72 statements were followed by the prompt *FALSE* (36 statements were positively and 36 statements were negatively phrased). Furthermore, the measure contained 60 catch trials (30 statements were positively and 30 statements were negatively phrased) where people needed to respond if they agreed or disagreed with a statement (??*TRUE/FALSE*??). Thus, the PEP consisted of 204 trials in total (144 prompt trials and 60 catch trials = 6 statements × 12 prompt (*TRUE*) + 6 statements × 12 prompt (*FALSE*) + 6 statements × 10 catch trials). Accuracy on the measure reflects the ratio of correctly responding to *TRUE/FALSE* prompts.

As in Study 1, we registered the area under the curve of participants’ responses and calculated participants’ time-normalized average trajectories across all trials. Greater deviation from the optimal trajectory indicates a smaller automatic tendency to agree (true) or disagree (false) with the presented item. We created the implicit score of intelligence mindset, using the method and code of Cummins and De Houwer’s^34^ experiments. The implicit score was coded in a way that higher scores represent more of a growth mindset.

#### Statistical analysis

In the results section, first we introduce the pre-registered analysis, testing the hypothesis with outcome-adjusted models (i.e., two-step models); second, we include the explicit scale in multiple regressions; third, we conduct hierarchical linear regressions to test the additive standardized effects of the implicit and explicit scores.

### Supplementary Information

Supplementary Information.

## Data Availability

We explain how we determined our sample sizes, pre-registered data exclusions, and all measures in the study. The studies were pre-registered at the following links: https://aspredicted.org/6xu2q.pdf; https://aspredicted.org/k9zr3.pdf. The code for data cleaning, analysis and visualizations as well as the materials can be found at the following link: https://osf.io/23npt/. The studies were approved by the institutional review board of the University of Vienna.
