# Supplementary material for: An implicit measure of growth mindset uniquely predicts post-failure learning behavior
Source: Sci Rep. 2024 Feb 14;14:3761. doi: 10.1038/s41598-024-52916-5 (PMC10867018; doi:10.1038/s41598-024-52916-5)
Supplement: Supplementary file 1 — Supplementary Information. [file 41598_2024_52916_MOESM1_ESM.docx]

An implicit measure of growth mindset uniquely predicts post-failure learning behavior: Supplementary materials

Kata Sik^1^, Jamie Cummins^2 3^, Veronika Job^1^

^1^Department of Occupational, Economic, and Social Psychology, University of Vienna, Austria; ^2^ Department of Experimental Clinical and Health Psychology, Ghent University, Belgium; ^3^ Institute of Psychology, University of Bern, Switzerland

**Author Note**

**ORCID:**Kata Sik: <https://orcid.org/0000-0002-0897-258X>;
Jamie Cummins: <https://orcid.org/0000-0002-9729-4900>**;**Veronika Job: <https://orcid.org/0000-0002-4983-1644>.

**Corresponding author**:
Veronika Job
^1^Department of Occupational, Economic, and Social Psychology, University of Vienna, Austria; 1010 Wächtergasse 1, Vienna, Austria
**Email**: [veronika.job@univie.ac.at](mailto:veronika.job@univie.ac.at)

**Study 1: Supplementary material**

Explicit IQ mindset:

Spinath, B., Stiensmeier-Pelster, J., Schöne, C., & Dickhäuser, O. (2002). *Skalen zur Erfassung der Lern- und Leistungsmotivation: SELLMO*. Hogrefe. <https://madoc.bib.uni-mannheim.de/42725/>

- Jeder besitzt ein bestimmtes Ausmaß an Intelligenz, das nicht verändert werden kann.
- Jeder besitzt ein bestimmtes Ausmaß an Intelligenz, das verändert werden kann.
- Wenn man neue Dinge lernt, bleibt die Intelligenz gleich.
- Wenn man neue Dinge lernt, verändert sich die Intelligenz.
- Intelligenz ist etwas, das kaum verändert werden kann.
- Intelligenz ist etwas, das verändert werden kann.

Self-efficacy adapted to the IQ tasks:

Pintrich, P. R., & De Groot, E. V. (2012). *Motivated strategies for learning questionnaire* [Data set]. American Psychological Association. <https://doi.org/10.1037/t09161-000>

- Im Vergleich zu anderen StudienteilnehmerInnen erwarte ich, dass ich gut abschneide.
- Ich erwarte, dass ich in dem folgenden Test über intellektuelle Fähigkeiten sehr gut abschneiden werde.
- Meine intellektuellen Fähigkeiten sind im Vergleich zu anderen StudienteilehmerInnen ausgezeichnet.
- Ich denke, ich werde im folgenden Test intellektueller Fähigkeiten eine hohe Punktzahl erhalten.
- Ich bin sicher, dass ich die Probleme und Aufgaben in diesem Test gut lösen kann.
- Verglichen mit anderen StudienteilnehmerInnen bin ich ein/e gute/r StudentIn.
- Ich weiß, dass ich in der Lage sein werde, die im Test gestellten Aufgaben zu verstehen.

Effort approach and effort avoidance goals:

Sik, K., & Job, V. (in preparation). Effort Importance and Effort Enjoyment in the Context of Math.

Bei diesem Test zu intellektuellen Fähigkeiten war mein Ziel,...

- … möglichst alle Aufgaben richtig zu lösen.
- … keine Fehler zu machen.
- **… meine Leistung von Aufgabe zu Aufgabe zu verbessern.**
- **...mich möglichst stark anzustrengen**
- **...mit voller Anstrengung mein Bestes zu geben.**
- **... mich möglichst wenig anzustrengen.**
- ... **zu vermeiden, dass ich mich zu sehr verausgabe.**

Controllability attributions:

Song, J., Kim, S., & Bong, M. (2020). Controllability Attribution as a mediator in the effect of mindset on achievement goal adoption following failure. *Frontiers in Psychology*, 10. <https://www.frontiersin.org/articles/10.3389/fpsyg.2019.02943>

- Meine Leistung in den Aufwärmübungen ist Folge meiner Anstrengung.
- Anstrengungen können sich ändern, wenn ich versuche, sie zu ändern.
- Anstrengung ist etwas, dass ich verändern kann.
- Meine Leistung in den Aufwärmübungen ist Folge meiner intellektuellen Fähigkeiten.
- Intellektuelle Fähigkeiten können sich ändern, wenn ich versuche, sie zu ändern.
- Intellektuelle Fähigkeit ist etwas, dass ich verändern kann.

Self-perceived talent:

Bauer, C., Job, V. & Hannover, B. (under review). Am I talented enough? Doubts about talent as a central mechanism in first-generation students’ academic disadvantage.

- Ich denke, dass ich begabt bin.
- Ich denke, dass ich talentiert bin.

Field-specific ability belief adapted to IQ tasks:

Leslie, S.-J., Cimpian, A., Meyer, M., & Freeland, E. (2015). Expectations of brilliance underlie gender distributions across academic disciplines. *Science*, 347(6219), 262–265. <https://doi.org/10.1126/science.1261375>

- Um bei Aufgaben wie einer Aufgabe zu intellektuellen Fähigkeiten Spitzenleistung erzielen zu können, muss man eine besondere Begabung haben, die man nicht erlernen kann.
- Wenn man bei Aufgaben wie einer Aufgabe zu intellektuellen Fähigkeiten wirklich erfolgreich sein will, reicht Anstrengung allein nicht aus; man muss eine angeborene Begabung oder Talent haben.
- Mit dem richtigen Maß an Anstrengung und Einsatz kann jede/r Spitzenleistungen bei Aufgaben wie einer Aufgabe zu intellektuellen Fähigkeiten erzielen.
- Bei Aufgaben wie einer Aufgabe zu intellektuellen Fähigkeiten sind Motivation und Konzentration die wichtigsten Erfolgsfaktoren; Begabung ist zweitrangig.
- Um bei Aufgaben wie einer Aufgabe zu intellektuellen Fähigkeiten Spitzenleistung erzielen zu können, muss man eine besondere Begabung haben, die man nicht erlernen kann.
- Wenn man bei Aufgaben wie einer Aufgabe zu intellektuellen Fähigkeiten wirklich erfolgreich sein will, reicht Anstrengung allein nicht aus; man muss eine angeborene Begabung oder Talent haben.
- Mit dem richtigen Maß an Anstrengung und Einsatz kann jede/r Spitzenleistungen bei Aufgaben wie einer Aufgabe zu intellektuellen Fähigkeiten erzielen.
- Bei Aufgaben wie einer Aufgabe zu intellektuellen Fähigkeiten sind Motivation und Konzentration die wichtigsten Erfolgsfaktoren; Begabung ist zweitrangig.

Effort enjoyment scale:

Sik, K., & Job, V. (in preparation). Effort Importance and Effort Enjoyment in the Context of Math.

- Ich strenge mich sehr gerne an.
- Ich arbeite gerne an einfachen Aufgaben.
- Ich hasse es, mich anzustrengen.
- Ich hasse es, an leichten Aufgaben zu arbeiten.
- Es macht Spaß, sich anzustrengen.
- Es macht Spaß, an leichten Aufgaben zu arbeiten.
- Sich anzustrengen ist ermüdend.
- Leichte Aufgaben sind langweilig.
- Während ich mich anstrenge, merke ich, dass mich das ärgert.
- Während ich eine leichte Aufgabe erledige, merke ich, dass mich das ärgert.
- Es fällt mir schwer, mich auf schwierige Aufgaben zu konzentrieren.
- Es fällt mir schwer, mich auf leichte Aufgaben zu konzentrieren.

Task enjoyment was created for the study:

- Wie gut hat Ihnen die Bearbeitung der Aufwärmübungen gefallen?
- Wie gut hat Ihnen die Bearbeitung des Haupttestes gefallen?

Achievement motivation:

Lang, J. W. B., & Fries, S. (2006). A revised 10-item version of the achievement motives scale. *European Journal of Psychological Assessment*, 22(3), 216–224. <https://doi.org/10.1027/1015-5759.22.3.216>

- Ich mag Situationen, in denen ich feststellen kann, wie gut ich bin.
- Wenn mir eine Aufgabe gestellt wird, die ich möglicherweise lösen kann, dann reizt es mich, sofort damit anzufangen.
- Situationen, in denen ich von meinen Fähigkeiten Gebrauch machen kann, machen mir Spaß.
- Mich reizen Situationen, in denen ich meine Fähigkeiten testen kann.
- Ich fühle mich zu Arbeiten hingezogen, in denen ich die Möglichkeit habe, meine Fähigkeiten zu prüfen.
- In etwas schwierigen Situationen, in denen viel von mir selbst abhängt, habe ich Angst zu versagen.
- Wenn mir ein Problem gestellt wird, das ich vielleicht lösen kann, dann reizt es mich, damit sofort anzufangen.
- Arbeiten, die ich nicht schaffen kann, machen mir Angst, auch dann, wenn niemand meinen Misserfolg merkt.
- Auch wenn niemand zuguckt, fühle ich mich in neuen Situationen ziemlich ängstlich.
- Wenn ich ein Problem nicht sofort verstehe, werde ich ängstlich.

Trait anxiety:

Poythress, N. G., Skeem, J. L., Weir, J., Lilienfeld, S. O., Douglas, K. S., Edens, J. F., & Kennealy, P. J. (2008). Psychometric properties of Carver and White’s (1994) BIS/BAS scales in a large sample of offenders. *Personality and Individual Differences*, 45(8), 732–737. <https://doi.org/10.1016/j.paid.2008.07.021>

- Kritik oder Tadel verletzt mich zutiefst.
- Ich habe Angst, Fehler zu machen.
- Ich fühle mich ziemlich besorgt oder aufgebracht, wenn ich denke oder weiß, dass jemand wütend auf mich ist.
- Wenn ich denke, dass etwas Unangenehmes passieren wird, dann werde ich normalerweise ziemlich angespannt.

**Exploratory moderator analysis:**

We conducted additional moderation analysis with all scales listed in this supplementary material, more specifically:

- Achievement motivation
- Trait anxiety
- Controllability attributions
- Effort enjoyment
- Field-specific ability beliefs
- Self-perceived talent
- Approach and avoidance goals
- Self-efficacy
- Explicit IQ mindset

The analysis and its output can be found at the following link: <https://osf.io/23npt/?view_only=72c6208140d5425b9d1addcc216640a9>, under ‘Exploratory Moderator Analysis’. None of the moderation analysis revealed any statistically significant results. However, it is important to note that we did not have enough power to find significant effects for moderation analyses. We designed our study for the pre-registered hypothesis. Thus, well-powered research would be necessary to prove whether there are any statistically meaningful moderation effects with these variables.

**Study 2: Supplementary material**

Explicit IQ mindset:

Spinath, B., Stiensmeier-Pelster, J., Schöne, C., & Dickhäuser, O. (2002). *Skalen zur Erfassung der Lern- und Leistungsmotivation: SELLMO*. Hogrefe. <https://madoc.bib.uni-mannheim.de/42725/>

- Jeder besitzt ein bestimmtes Ausmaß an Intelligenz, das nicht verändert werden kann.
- Jeder besitzt ein bestimmtes Ausmaß an Intelligenz, das verändert werden kann.
- Wenn man neue Dinge lernt, bleibt die Intelligenz gleich.
- Wenn man neue Dinge lernt, verändert sich die Intelligenz.
- Intelligenz ist etwas, das kaum verändert werden kann.
- Intelligenz ist etwas, das verändert werden kann.

Self-efficacy adapted to the IQ tasks:

Pintrich, P. R., & De Groot, E. V. (2012). *Motivated strategies for learning questionnaire* [Data set]. American Psychological Association. <https://doi.org/10.1037/t09161-000>

- Im Vergleich zu anderen StudienteilnehmerInnen erwarte ich, dass ich gut abschneide.
- Ich erwarte, dass ich in dem folgenden Test über intellektuelle Fähigkeiten sehr gut abschneiden werde.
- Meine intellektuellen Fähigkeiten sind im Vergleich zu anderen StudienteilehmerInnen ausgezeichnet.
- Ich denke, ich werde im folgenden Test intellektueller Fähigkeiten eine hohe Punktzahl erhalten.
- Ich bin sicher, dass ich die Probleme und Aufgaben in diesem Test gut lösen kann.
- Verglichen mit anderen StudienteilnehmerInnen bin ich ein/e gute/r StudentIn.
- Ich weiß, dass ich in der Lage sein werde, die im Test gestellten Aufgaben zu verstehen.

Metacognition:

Pintrich, P. R., & De Groot, E. V. (1990). Motivational and self-regulated learning components of classroom academic performance. *Journal of educational psychology*, *82*(1), 33

- Ich übersehe oft wichtige Punkte, weil ich an etwas anderes denke.
- Wenn ich etwas lese, dann erfinde ich Fragen dazu, um mich besser zu fokussieren.
- Wenn mich etwas verwirrt, dann lese ich es zum besseren Verständnis noch einmal durch.
- Wenn etwas zu schwer zu verstehen ist, dann ändere ich meine Herangehensweise.
- Bevor ich etwas gründlich lese, überfliege ich es zunächst, um den Aufbau zu verstehen.
- Ich stelle mir selbst Fragen, um sicherzugehen, dass ich alles verstanden habe.
- Nachdem ich etwas gelesen habe, weiß ich oft nicht mehr worum es ging.
- Ich denke darüber nach, was ich von einer Thematik lernen möchte, anstatt sie nur durchzulesen.
- Wenn ich für einen Kurs lerne, dann überlege ich mir, welche Konzepte ich nicht gut verstehe.
- Ich setzte mir selbst Ziele, um meine Tätigkeiten besser zu lenken.

Failure mindset (translated to German):

Haimovitz, K., & Dweck, C. S. (2016). What predicts children’s fixed and growth intelligence mind-sets? Not their parents’ views of intelligence but their parents’ views of failure. *Psychological science*, *27*(6), 859-869.

- Misserfolge zu erleben fördert das Lernen.
- Misserfolge zu erleben steigert meine Leistung.
- Misserfolge zu erleben verhindert mein Wachsen.
- Die Konsequenzen von Misserfolgen sind negativ und sollten vermieden werden.

Task enjoyment was created for the study:

- Wie gut hat Ihnen die Bearbeitung der Aufwärmübungen gefallen?
- Wie gut hat Ihnen die Bearbeitung des Haupttestes gefallen?

**Exploratory moderator analysis:**

We conducted additional moderation analysis with all scales listed in this supplementary material, more specifically:

- Metacognition
- Failure mindset
- Self-efficacy
- Explicit IQ mindset

The analysis and its output can be found at the following link: <https://osf.io/23npt/> under ‘Exploratory Moderator Analysis’. None of the moderation analysis revealed any statistically significant results. However, it is important to note that we did not have enough power to find significant effects for moderation analyses. We designed our study for the pre-registered hypothesis. Thus, well-powered research would be necessary to prove whether there are any statistically meaningful moderation effects with these variables.
